# The Value of Definite Therapeutical Combination

**Published:** 1884-01

**Authors:** 


### SELECTIONS.

### The Value of Definite Therapeutical Combinations.

A singularly unfounded prejudice against definite combinations of therapeutical agents--in other words, typical formulæ and recipes--has of late years grown up in the profession. The origin of this prejudice is, doubtless, the mechanical and unreflecting use which some men make of a formula, copying it out, and applying it promiscuously whenever the disease occurs for which it is recommended. That, of course, is always to be condemned. The proper use of a formula is as a typical combination, to be varied as the age, condition, and idiosyncrasy of the patient require. Thus used, a judicious recipe, founded on a scientific acquaintance with drugs, and supported by intelligent clinical experience, is just as valuable as the discovery of a new agent in therapeutics--often much more so.

Let us rehearse the advantages which a really good combination of the kind presents.

In the first place, *greater efficiency.* All intelligent therapeutists now acknowledge the *synergic* action of drugs—that the particular effect we desire from a drug can be obtained more certainly, more potently, and with fewer unpleasant accidents, by combining it with some other drugs. The old-fasioned Dover's Powder is a remarkable example of this, which we all know from experience, but cannot easily explain. Very many other instances are adduced by Bartholow and other late writers.

In the second place, we can, by combinations, often *obtain results not obtainable by simple agents.* Thus in not a few cases of anæmia the result of administering iron alone is very unsatisfactory; while a combination of this agent, say with ammonia, will bring about immediate improvement. Many such combinations are not, properly speaking, synergistic; the action of the adjuvant does not directly increase the effect of the main ingredient, but simply gives it a chance to exert its ordinary effect.

The *prevention of unpleasant effects* is another end often gained by a skillful combination. Every one knows what disagreeable consequences on many constitutions has the administration of opium in any form, of iodide of potash, and of many other extremely useful drugs. It is scarcely too much to say that skillful combinations will secure all their beneficial effects and avoid all their detrimental or annoying consequences.

Nor is it by any means to be despised that a judicious formula enables us to make our remedies *palatable*. From a neglect of this, many a family becomes dissatisfied with their physician, and seeks one who pays greater attention to the art of making agreeable recipes. And these particular families are precisely those willing to pay the best fees.

*Permanency and uniformity* in a medicinal combination are other advantages often secured by a good recipe. They are often of no little weight. A few years ago a woman in this city died from a tonic mixture of strychnia, having taken all the strychnia in a six-ounce mixture in the last teaspoonful! Had her physician known how to combine it properly, this would not have occurred, and he would have saved his reputation. Chloral and many other drugs rapidly deteriorate in some combinations, and are very permanent in others. The study of formulas based on science and observation is peculiarly demanded for the proper administration of such agents.

The *avoidance of incompatibility* is of growing importance, through the introduction of so many chemical drugs. Physiological incompatibility, the doctrine of clinical antagonism, also comes up for consideration. Incompatibles may render each other inert, or actively poisonous and dangerous. Their list is a long one, and it must be learned by heart, and never forgotten, except where an already well-tried formula is followed.

These very brief considerations show how great is the value of definite therapeutic combinations which have stood the test of experience.

